# SlMYB72 affects pollen development by regulating autophagy in tomato

**DOI:** 10.1093/hr/uhac286

**Published:** 2022-12-29

**Authors:** Mengbo Wu, Qiongdan Zhang, Guanle Wu, Lu Zhang, Xin Xu, Xiaowei Hu, Zehao Gong, Yulin Chen, Zhengguo Li, Honghai Li, Wei Deng

**Affiliations:** Key Laboratory of Plant Hormones and Development Regulation of Chongqing, School of Life Sciences, Chongqing University, Chongqing 400044, China; Center of Plant Functional Genomics, Institute of Advanced Interdisciplinary Studies, Chongqing University, 401331 Chongqing, China; Key Laboratory of Plant Hormones and Development Regulation of Chongqing, School of Life Sciences, Chongqing University, Chongqing 400044, China; Center of Plant Functional Genomics, Institute of Advanced Interdisciplinary Studies, Chongqing University, 401331 Chongqing, China; Key Laboratory of Plant Hormones and Development Regulation of Chongqing, School of Life Sciences, Chongqing University, Chongqing 400044, China; Center of Plant Functional Genomics, Institute of Advanced Interdisciplinary Studies, Chongqing University, 401331 Chongqing, China; Department of Horticulture and Landscape Architecture, Oklahoma State University, Stillwater, OK 74078, USA; Key Laboratory of Plant Hormones and Development Regulation of Chongqing, School of Life Sciences, Chongqing University, Chongqing 400044, China; Center of Plant Functional Genomics, Institute of Advanced Interdisciplinary Studies, Chongqing University, 401331 Chongqing, China; Key Laboratory of Plant Hormones and Development Regulation of Chongqing, School of Life Sciences, Chongqing University, Chongqing 400044, China; Center of Plant Functional Genomics, Institute of Advanced Interdisciplinary Studies, Chongqing University, 401331 Chongqing, China; Key Laboratory of Plant Hormones and Development Regulation of Chongqing, School of Life Sciences, Chongqing University, Chongqing 400044, China; Center of Plant Functional Genomics, Institute of Advanced Interdisciplinary Studies, Chongqing University, 401331 Chongqing, China; Key Laboratory of Plant Hormones and Development Regulation of Chongqing, School of Life Sciences, Chongqing University, Chongqing 400044, China; Center of Plant Functional Genomics, Institute of Advanced Interdisciplinary Studies, Chongqing University, 401331 Chongqing, China; Key Laboratory of Plant Hormones and Development Regulation of Chongqing, School of Life Sciences, Chongqing University, Chongqing 400044, China; Center of Plant Functional Genomics, Institute of Advanced Interdisciplinary Studies, Chongqing University, 401331 Chongqing, China; Key Laboratory of Plant Hormones and Development Regulation of Chongqing, School of Life Sciences, Chongqing University, Chongqing 400044, China; Center of Plant Functional Genomics, Institute of Advanced Interdisciplinary Studies, Chongqing University, 401331 Chongqing, China; Key Laboratory of Plant Hormones and Development Regulation of Chongqing, School of Life Sciences, Chongqing University, Chongqing 400044, China; Center of Plant Functional Genomics, Institute of Advanced Interdisciplinary Studies, Chongqing University, 401331 Chongqing, China

## Abstract

The formation and development of pollen are among the most critical processes for reproduction and genetic diversity in the life cycle of flowering plants. The present study found that *SlMYB72* was highly expressed in the pollen and tapetum of tomato flowers. Downregulation of *SlMYB72* led to a decrease in the amounts of seeds due to abnormal pollen development compared with wild-type plants. Downregulation of *SlMYB72* delayed tapetum degradation and inhibited autophagy in tomato anther. Overexpression of *SlMYB72* led to abnormal pollen development and delayed tapetum degradation. Expression levels of some autophagy-related genes (ATGs) were decreased in *SlMYB72* downregulated plants and increased in overexpression plants. SlMYB72 was directly bound to ACCAAC/ACCAAA motif of the *SlATG7* promoter and activated its expression. Downregulation of *SlATG7* inhibited the autophagy process and tapetum degradation, resulting in abnormal pollen development in tomatoes. These results indicated SlMYB72 affects the tapetum degradation and pollen development by transcriptional activation of *SlATG7* and autophagy in tomato anther. The study expands the understanding of the regulation of autophagy by SlMYB72, uncovers the critical role that autophagy plays in pollen development, and provides potential candidate genes for the production of male-sterility in plants.

## Introduction

Pollen formation and development are necessary for the reproduction of flowering plants and consist of several distinct stages [1]. Pollen grains are developed from microspore mother cells in the microsporangium of an anther. Each microspore mother cell can form a microspore tetrad (four haploid microspores) through meiotic division. When the anther matures, the microspores dissociate and develop into pollen grains [2]. The development of pollen could be influenced by many factors, such as tapetum irregularity [3–6], cytoskeleton alteration [7, 8], auxin metabolism aberration [9, 10], altered sugar utilization [6, 11], and reactive oxygen species [12, 13]. Among all these factors the tapetum plays an important role in the regulation of pollen development [14].

Tapetum is the innermost layer of the anther and adheres to the microspores. The tapetum provides various enzymes, proteins, lipids, starch, sporopollenin, and other molecules required for pollen development [1, 15, 16]. An adequately programmed cell death (PCD) is necessary for the degradation of tapetum, which for pollen development and microspores release [17, 18]. Many transcription factors (TFs) affect pollen development by regulating the tapetum PCD. The knocking out of the *AtMYB103* gene in Arabidopsis leads to defective tapetal cell wall degradation, pollen development, and pollen exine formation [19]. Mutants of Arabidopsis *MYB33*, *MYB65*, and rice *GAMYB* genes result in a loss of tapetum PCD and abnormal pollen development [20–22]. Arabidopsis MYB80 regulates the tapetal PCD and pollen development by direct binding to pectin methylesterase, glyoxal oxidase, and aspartic protease genes [3]. A putative *bHLH* gene affects the tapetum development and pollen formation in tomatoes [23].

Macroautophagy (autophagy) is involved in plant growth and development and plays critical roles in many abiotic and biotic stress responses [24]. In normal circumstances, autophagy remains at a base level to degrade cytoplasmic components and maintain homeostasis in cells. Previous research has well documented that autophagy loss reduces seed yields and causes the senescence of premature leaves [25–27], but an increased level of autophagy promotes growth, seed yields, and nitrogen remobilization [28, 29]. Autophagy is upregulated and recycles cellular material and nutrients to promote plant survival during senescence or stress response [24, 30, 31]. Autophagy is involved in cell death during tapetum degradation [32]. In tobacco, the overexpression of Arabidopsis *ATG6*/*Beclin1* genes in tapetum results in sterility, and excessive autophagy increases the PCD and abortion of microsporogenesis [32, 33]. The rice autophagy mutant OsATG7 exhibits delay of the tapetum layer’s degradation and leads to sporophytic male sterility [34, 35]. High temperatures lead to tapetal PCD abortion and damage pollen development, but autophagy mitigates the injury in pollen development in Arabidopsis [36].

The R2R3MYB is one of the most prominent families of TFs and is involved in plant development, metabolism, and stress responses. We have reported that SlMYB72 is a typical R2R3 MYB family TF and is involved in the accumulation of pigment in tomato fruits [37]. In the present study, we found that SlMYB72 affected the tapetum degradation and pollen development in tomatoes. SlMYB72 affected SlATG7 expression and autophagy process in tomato anthers. SlATG7 also regulated the tapetum degradation and pollen development in tomatoes. The results strongly suggest that SlMYB72 affects pollen development by regulating autophagy in tomatoes and uncovers the important role of autophagy in tomato development.

## Results

### *SlMYB72* highly expresses in pollen and tapetum of tomato

**Figure 1:**
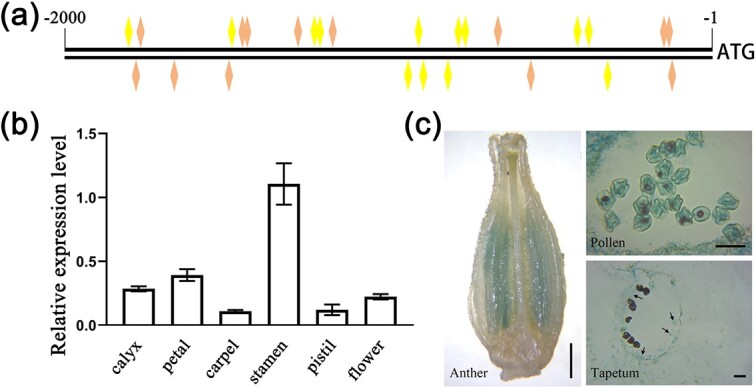
Analysis of promoter sequence and expression patterns of *SlMYB72* gene in tomatoes. **a** Scheme of the *SlMYB72* gene promoter. Yellow quadrilateral, POLLEN1LELAT52 pollen-specific activation element (AGAAA). Orange quadrilateral, late pollen gene g10-related element (GTGA). **b** RT-qPCR analysis of *SlMYB72* expression level in the different organs of opening flowers. The data are means ± SD (four biological replicates). **c** Analysis of *SlMYB72* expression pattern. Left scale bar = 1 mm, right scale bar = 35 μm.

Our previous study has shown that SlMYB72 is a typical R2R3 MYB family transcription factor. In this study, the *SlMYB72* promoter sequence was submitted to a public database (http://www.dna.affrc.go.jp/PLACE/signalup.html) to search for the *cis*-acting element. The *SlMYB72* promoter contains pollen-specific elements POLLEN1LELAT52 (AGAAA) and GTGANTG10 (GTGA) (Fig. 1a). This result indicated that *SlMYB72* might be involved in the pollen formation.

The expression level of *SlMYB72* in flower tissue was precisely analysed. RT-qPCR data showed that the *SlMYB72* gene was expressed weekly in the calyx, petal, carpel, and pistil, but expressed strongly in the stamen (Fig. 1b). Glucuronidase (GUS) staining of plant tissues was used to analyse the SlMYB72 expression pattern. As shown in Fig. 1c, expression of the GUS gene driven by the *SlMYB72* promoter was found in the stamen, especially in pollen and tapetum. These results indicated that *SlMYB72* has a tissue-specific expression in pollen and tapetum.

### Downregulation of *SlMYB72* affects the pollen development in tomatoes

Our previous study generated six *SlMYB72* downregulated transgenic plants (Lines 3, 5, 8, 9, 10, and 11). The RNAi-SlMYB72 lines 9, 10, and 11 exhibited a 50–60% decrease in *SlMYB72* expression and about a 50% decrease in seed count compared with the WT plant, but Lines 3, 5, and 8 with the 80–90% decrease in *SlMYB72* expression levels did not produce seeds (Fig. S1, see online supplementary material). This phenotype indicated that downregulation of the expression of SlMYB72 inhibited seed formation.

**Figure 2:**
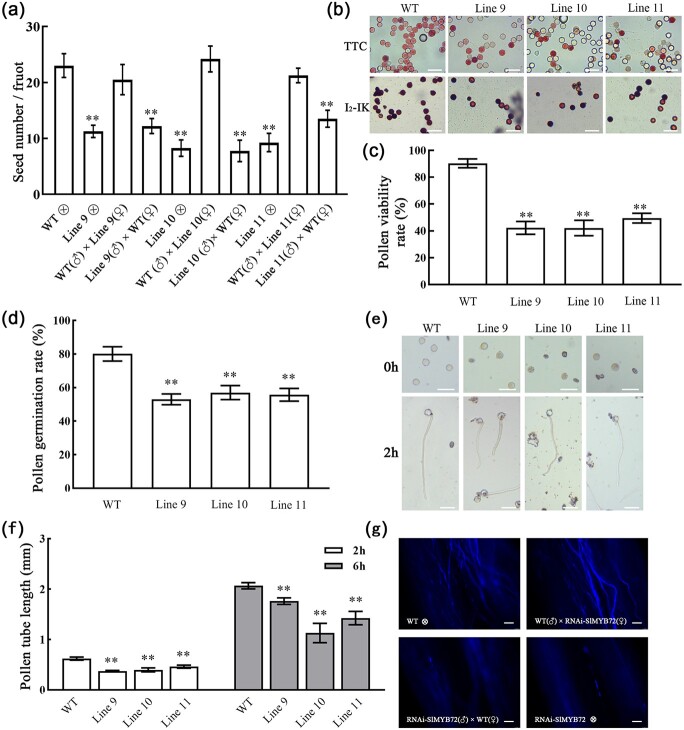
Seed count and pollen viability analysis in RNAi-SlMYB72 plants. **a** Fruit seed number of self- or cross-pollinated RNAi-SlMYB72 and WT plants. Seed number of self-pollinated plants (WT $ \otimes $, Line9 $ \otimes $, Line10 $ \otimes $, and Line11 $ \otimes $), WT pistil pollinated with RNAi-SlMYB72 pollen [line9 (♂) × WT (♀), line10 (♂) × WT (♀) and line11 (♂) × WT (♀)], RNAi-SlMYB72 pistil pollinated with WT pollen [WT (♂) × line9 (♀), WT (♂) × line10 (♀) and WT (♂) × line11 (♀)]. **b** Pollen viability. Pollens were stained with TTC and I2-KI. Viable pollens were stained red with TTC staining. Viable pollens were stained dark with I_2_-KI staining. Scale bar = 50 μm. **c** Statistics analysis of pollen vitality by I_2_-KI staining. **d***In vitro* pollen germination rate. Pollens were incubated on germination media at 22°C for 1 h. Scale bar = 50 μm. **e**, **f***In vitro* pollen tube growth. Pollens were incubated on germination media at 22°C for 2 and 6 h. Data are means ± SD (ten biological replicates). Asterisks represent significant differences between WT and RNAi-SlMYB72 plants (Student’s *t*-test, ^*^P < 0.05 and ^**^P < 0.01). Scale bar = 50 μm. **g** Fluorescence observation of pollen tube growth in the style of pistil after pollination (2 DAP). Scale bar = 50 μm.

To examine whether the downregulation of *SlMYB72* affected female or male fertility, a cross-assay was performed. When we used the RNAi-SlMYB72 plant as the female parent to cross with the male parent from the WT plant, the seed count of the hybrid was similar to the self-pollinating WT plant, but the seed number was reduced by half when we used the RNAi-SlMYB72 plant as the male parent to cross with the WT female parent (Fig. 2a). This result indicated that reducing the number of seeds might be due to decreased pollen viability in RNAi-SlMYB72 flowers. Then the pollen viability was tested by I_2_-KI and TTC staining. About 90% of the pollen in WT exhibited viability, while about 40%–50% of the pollen grains had viability in RNAi-SlMYB72 plants (Fig. 2b and c). We further investigated the germination and growth of pollen in RNAi-SlMYB72 and WT plants. The germination rate of WT pollen was 80%, but only about 52%–56% in RNAi-SlMYB72 plants (Fig. 2d). The RNAi-SlMYB72 plants also showed decreased pollen growth compared with the WT plants (Fig. 2e and f). Moreover, pollen growth in style was observed after pollination. When the RNAi-SlMYB72 plant as the female parent was used to cross with the male parent from the WT plant, the fluorescent signal of pollen tubes in the style was clearly observed, which was similar to the self-pollinating WT plants, but the fluorescent signal of pollen tubes was not clearly observed when we used the RNAi-SlMYB72 plant as the male parent to cross with the WT female parent (Fig. 2g). The data indicated that down-regulation of *SlMYB72* affects the pollen development in tomatoes.

**Figure 3:**
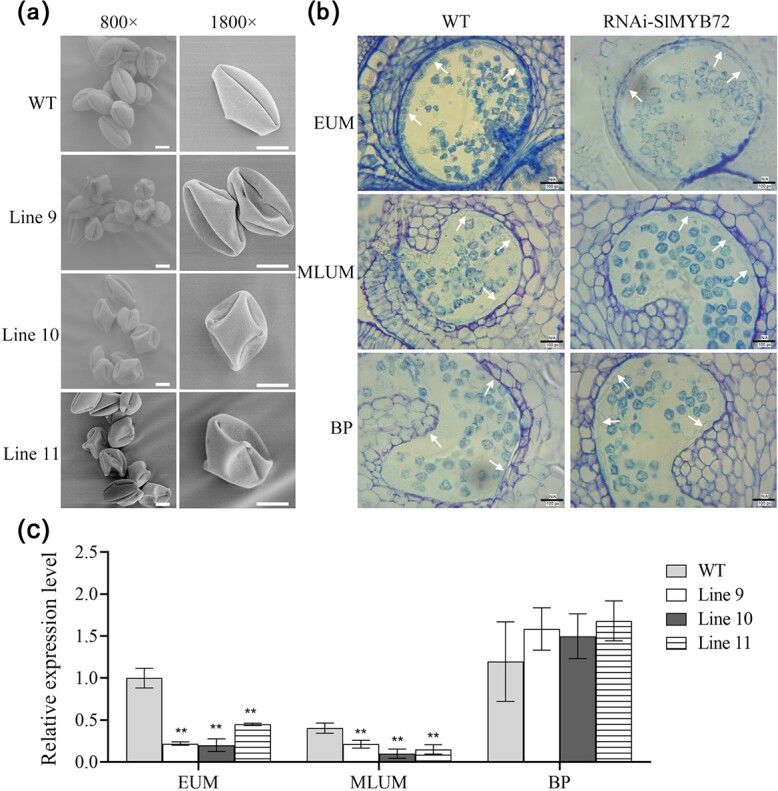
Histological analysis of anthers in RNAi-SlMYB72 and WT plants. **a** Scan electron microscope observation of pollens in WT and RNAi-SlMYB72 flower at full bloom stage. Scale bars = 10 μm. **b** Anatomical analysis of WT and RNAi-SlMYB72 anthers at different development stages. EUM, MLUM, and BP represent the stages of early uninucleate microspore, middle, and later uninucleate microspore, and binucleate pollen. Arrows indicate tapetum. Scale bar = 30 μm. **c** RT-qPCR analysis of the expression levels of *SlMYB72* at different development stages. The data are means ± SD (four biological replicates). Asterisks represent significant differences between WT and RNAi-SlMYB72 plants (Student’s *t*-test, ^*^P < 0.05 and ^**^P < 0.01). Scale bar = 50 μm.

The morphology of mature pollen was analysed by using scanning electron microscopy (SEM). The pollens of RNAi-SlMYB72 plants were shriveled and irregular in shape compared with the WT pollens (Fig. 3a). A semi-thin section was used to investigate the anatomic structure of mature anthers. The tapetum of the RNAi-SlMYB72 plant was the same as the WT plant at the uninucleate microspore stage. The WT tapetum has disappeared completely, but the tapetum of the RNAi-SlMYB72 plant still had a complete cell morphology at the binucleus microspore stage (Fig. 3b). The expression of MYB72 gene in transgenic lines and WT at the different stage was examined by RT-qPCR. The expression level of *SlMYB72* was significantly lower than that of the control group in the EUM and MLUM stages, but there was no significant difference in the BP stage (Fig. 3c). This result indicated that downregulation of *SlMYB72* delayed the tapetum degeneration in tomato anthers.

### Downregulation of the *SlMYB72* gene inhibits the autophagy in tomato anthers

**Figure 4:**
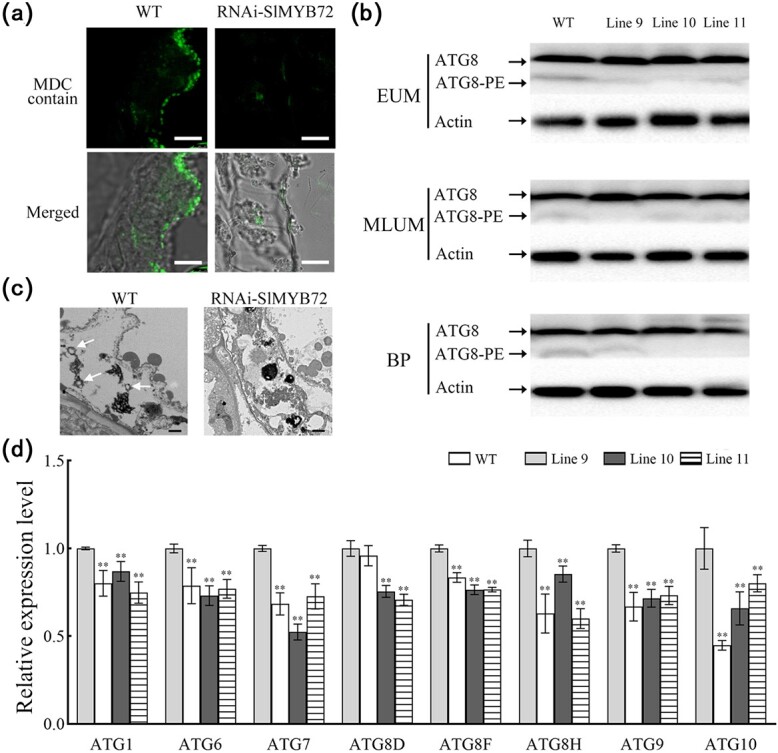
Analysis of autophagy in WT and RNAi-SlMYB72 anther. **a** Autophagosomes stained with MDC in the tapetum of RNAi-SlMYB72 and WT plants. The tissue was induced with exogenous rapamycin. The autophagosomes stained with MDC are shown in green fluorescence. Scale bars = 5 μm. **b** ATG8 protein levels in RNAi-SlMYB72 and WT anthers. ATG8-PE and ATG8 are the lipidated and nonlipidated ATG8 forms, respectively. Internal control is actin. EUM, MLUM, and BP represent the stages of early uninucleate microspore, middle, and later uninucleate microspore, and binucleate pollen. **c** TEM observation of autophagosomes in the tapetum of RNAi-SlMYB72 and WT plants. White arrows indicate autophagosomes. Scale bars = 1 μm. **d** Expression of *ATGs* in the WT and RNAi-SlMYB72 anthers during MLUM stage. RT-qPCR was used to analyse the expression levels. Data are means ± SD (four biological replicates). Asterisks represent significant differences (Student’s *t*-test, ^*^P < 0.05 and ^**^P < 0.01).

It has been reported that autophagy is involved in tapetum degeneration and pollen development [35, 38]. We further analysed the autophagy process in the anther of RNAi-SlMYB72 plant in current research. Fluorescent dye monodansyl cadaverine (MDC) staining was used to analyse autophagosome occurrence in RNAi-SlMYB72 anthers. Compared with WT anther, the number of punctate fluorescent signals in RNAi-SlMYB72 anther was significantly reduced when autophagy was induced with exogenous rapamycin (Fig. 4a). To further confirm the result of MDC staining, western blotting (WB) was applied to explore the formation of ATG8-phosphatidylethanol-amine (ATG8-PE) conjugates as autophagy marker with an anti-ATG8a antibody. The ATG8-PE band was less abundant in RNAi-SlMYB72 anthers than that of WT anthers, especially at the early uninucleate microspore and binucleate stages (Fig. 4b). Then transmission electron microscopy (TEM) was used to study the autophagosome occurrence in RNAi-SlMYB72 anthers. Some autophagosome vesicles in the WT anthers were detected, while the autophagosome vesicles of RNAi-SlMYB72 anthers were hardly detected under the conditions of inducing autophagy (Fig. 4c). The results demonstrated that the downregulation of *SlMYB72* inhibited the autophagy process in tomato anthers.

The expression levels of autophagy-related genes (*ATGs*) were further analysed in RNAi-SlMYB72 anthers. RT-qPCR data showed seven *ATG* genes’ expression levels, including *SlATG1*, *SlATG6*, *SlATG7*, *SlATG8D*, *SlATG8F*, *SlATG8H*, *SlATG9*, and *SlATG10* were decreased in the anthers of RNAi-SlMYB72 plants (Fig. 4d).

### Overexpression of the *SlMYB72* gene results in abnormal pollen development and promoted tapetum degradation in tomato anthers

**Figure 5:**
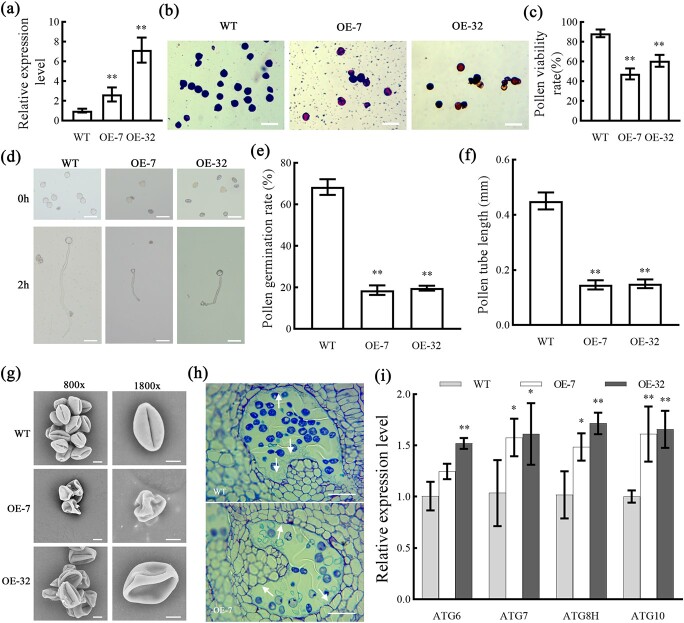
Analysis of pollen development and tapetum degradation in OE-SlMYB72 anthers. **a** Expression of the *SlMYB72* gene in the WT and OE-SlMYB72 plants. RT-qPCR was used to analyse the expression levels. Data are means ± SD (four biological replicates). Asterisks represent significant differences (Student’s *t*-test, ^**^P < 0.01). **b**, **c** Pollen viability. Pollens were stained with I2-KI. Viable pollens were stained dark with I_2_-KI staining. Scale bar = 50 μm. **d**, **e**, **f***In vitro* pollen germination and pollen tube growth. Pollens were incubated on germination media at 22°C for 2 h. Scale bar = 50 μm. Data are means ± SD (ten biological replicates). **g** Scan electron microscope observation of pollens in WT and OE-SlMYB72 flower at full bloom stage. Scale bars = 10 μm. **h** Anatomical analysis of WT and OE-SlMYB72 anthers at MLUM (middle and later uninucleate microspore) stages. Scale bar = 50 μm. **i** Expression of *ATGs* in the WT and OE-SlMYB72 anthers during MLUN stage. RT-qPCR was used to analyse the expression levels. Data are means ± SD (four biological replicates). Asterisks represent significant differences (Student’s *t*-test, ^*^P < 0.05 and ^**^P < 0.01).

To further analyse the function of *SlMYB72* in pollen development, two SlMYB72 overexpression lines (OE-SlMYB72) corresponding to independent transformation events were produced. RT-PCR analysis showed that the SlMYB72 gene expression level was increased in the OE-SlMYB72 plants (Fig. 5a; Fig. S3, see online supplementary material). The pollen viability in OE-SlMYB72 flowers was analysed by I_2_-KI stain. About 90% of the pollen in WT exhibited viability, while about 50%–60% of the pollen grains had viability in OE-SlMYB72 plants (Fig. 5b and c). The germination rate of pollen and the growth of pollen tubes in the OE-SlMYB72 plants were further detected (Fig. 5d–f). The pollen germination rate was about 70% in the WT plants, while only 20% in OE-SlMYB72 plants (Fig. 5e). The pollen tube length was tested after culturing for 2 hours. The length of the pollen tube reached 0.45 mm in the WT plants, while the length of the pollen tube was only 0.16 mm in OE-SlMYB72 plants (Fig. 5f). The results indicated that the pollen viability and pollen germination rate were significantly affected in the OE-SlMYB72 plants, which are similar to RNAi-SlMYB72 plants.

SEM was performed to analyse the morphology of mature pollen. Most of the pollen had intact morphology in the WT plants, while the pollen collapsed and was shrunken in the OE-SlMYB72 plants (Fig. 5g). The anatomic structure of mature anthers was analysed by a semi-thin section. The OE-SlMYB72 tapetum has disappeared completely, but the tapetum of the WT still had a lot of residuals at the middle and later uninucleate microspore stage (Fig. 5h). This result indicated that overexpression of SlMYB72 triggered premature tapetum degradation and pollen abortion. The expression levels of autophagy-related genes in OE-SlMYB72 plants were further investigated. The result of RT-qPCR indicated that four *ATG* genes’ expression levels were significantly increased in the anthers of OE-SlMYB72 plants, including *ATG6*, *ATG7*, *ATG8h*, and *ATG10* genes (Fig. 5i).

### SlMYB72 directly targets *SlATG7* genes and increases their expression

**Figure 6:**
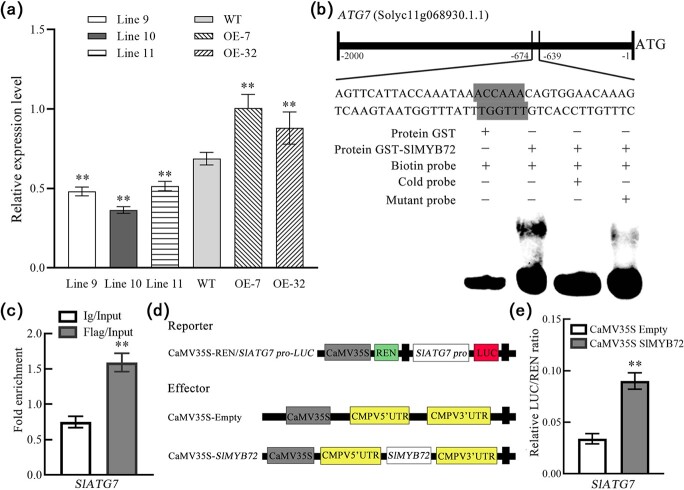
SlMYB72 targets the *SlATG7* promoter and increases its expression. **a** RT-qPCR analysis of the expression level of *SlATG7* in RNAi-SlMYB72 (Line 9, 10, 11), OE-SlMYB72 (OE-7, −32), and WT plants. Data are means ± SD (four biological replicates). MLUM represents the stages of the middle and later uninucleate microspores. **b** EMSA indicating direct binding of SlMYB72 to *SlATG7* promoters. Biotin-labeled DNA probes containing AC-rich motifs or mutated motifs were incubated with GST-SlMYB72. The protein-DNA complexes were separated using 6% (w/v) polyacrylamide gel electrophoresis. **c** ChIP-qPCR indicating direct binding of SlMYB72 to the *SlATG7* gene. Data are means ± SD (four biological replicates). **d** Vector diagrams of dual-luciferase reporter assay. **e** Dual-luciferase reporter assay indicating SlMYB72 increases the expression of the *SlATG7* gene. *Agrobacterium tumefaciens* carrying the effector and reporter vectors were infiltrated into tobacco leaves. LUC and REN activities were analysed. Data are means ± SD (six biological replicates). Asterisks represent significant differences (Student’s *t*-test, ^*^P < 0.05 and ^**^P < 0.01).

*OsATG7* has been reported to regulate tapetum degeneration and pollen development in rice [35]. RT-qPCR was performed to analyse the expression of *SlATG7* in MLUM stage. The expression of *SlATG7* in the RNAi-SlMYB72 plants was significantly lower than that in the WT plants, while it was significantly higher in the OE-SlMYB72 transgenic plant than that in the WT plants (Fig. 6a; Fig. S3, see online supplementary material). Promoter analysis found that the *SlATG7* promoter has an AC-rich element, an R2R3-MYB binding motif. An electrophoretic mobility shift assay (EMSA) was carried out to explore the direct binding of the SlMYB72 protein to the *SlATG7* gene. The recombinant proteins of SlMYB72 and GST (GST-SlMYB72) were successfully purified. The GST-SlMYB72 recombinant protein targeted the biotin-labeled probes which contained an AC-rich motif derived from the promoter of the *SlATG7* gene and produced a mobility shift. Unlabeled fragment of *SlATG7* promoter as a competitor abolished the gel mobility shift. But no mobility shift bands were detected when the probes were incubated with only GST (Fig. 6b). The EMSA result revealed that the SlMYB72 directly targeted the AC-rich element of the *SlATG7* promoter. Chromatin immunoprecipitation (ChIP) qPCR was carried out to verify the interaction between SlMYB72 and *SlATG7* promoter *in vivo. SlATG7* promoter region containing the AC-rich motif was enriched when FLAG antibodies were used, but not enriched when nonspecific antibodies (IgG) were used (Fig. 6c), which indicated that the SlMYB72 targeted the *SlATG7* promoter. A transient dual-luciferase reporter assay was performed to determine the regulation of *SlATG7* by SlMYB72. Overexpression of the SlMYB72 effectively increased the activity of luciferase driven by the *SlATG7* promoter, compared with the control (pEAQ) (Fig. 6d and e). The results further proved that SlMYB72 directly targeted the *SlATG7* promoter and increased gene expression.

### SlATG7 is involved in pollen development and seed formation

**Figure 7:**
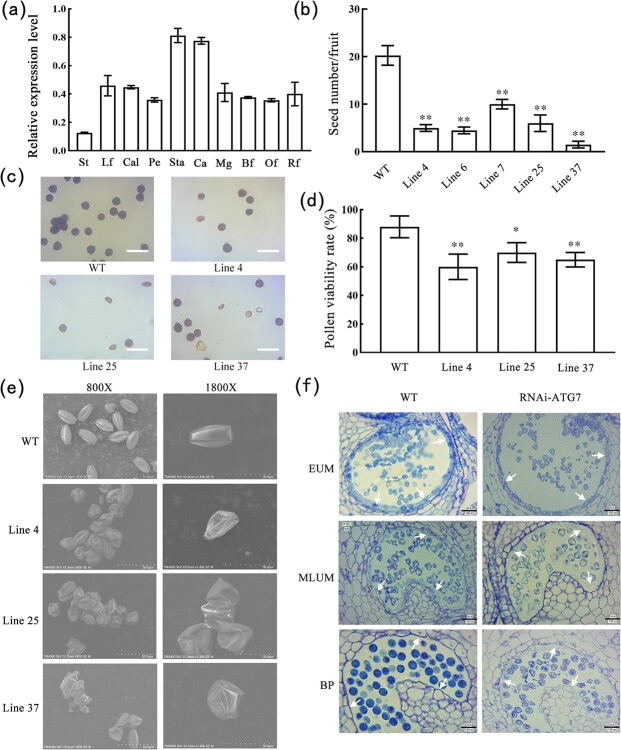
Seed count and pollen viability analysis of RNAi-SlATG7 and WT plants. **a** RT-qPCR analysis of the expression level of *SlATG7* in different organs. Data are means ± SD (four biological replicates). St, Lf, Cal, Pe, Sta, Ca, Mg, Bf, Of, and Rf represent Stem, Leaf, Calyx, Petal, Stamen, Carpel, mature green fruit, breaker fruit, orange fruit, and red fruit, respectively. **b** Seed number per fruit of RNAi-SlATG7 and WT plants. Data are means ± SD (six biological replicates). **c** Viability of mature pollen. I_2_-KI was used to stain pollens. Scale bar = 50 μm. **d** Mature pollen viability rate. Pollen was incubated on germination media for 1 hour at 22°C. Scale bar = 50 μm. **e** SEM observation of pollens in WT and RNAi-SlATG7 flowers at full bloom stage. Scale bars = 10 μm. **f** Anatomical analysis of WT and RNAi-SlATG7 anthers at different development stages. EUM, MLUM, and BP represent the stages of early uninucleate microspore, middle and later uninucleate microspore, and binucleate pollen. Arrows indicate tapetum. Scale bar = 30 μm. Asterisks represent significant differences (Student’s *t*-test, ^*^P < 0.05 and ^**^P < 0.01).

SlATG7 protein contains 716 amino acid residues including an N-terminal domain of ubiquitin-like modifier-activating enzyme ATG7 (ATG7-N) and a NAD/FAD-binding domain (ThiF) (Fig. S2a, see online supplementary material). Phylogenetic analysis revealed that the SlATG7 was close to NtATG7 and AtATG7 (Fig. S2b, see online supplementary material). The expression pattern of the *SlATG7* gene was analysed using a public database (http://tomexpress.toulouse.inra.fr/query). The *SlATG7* gene was expressed in all of the tomato plants, but was highest in the tomato flower tissues (Fig. S4, see online supplementary material). Then RT-qPCR was performed to obtain further insights into the expression pattern, and the result showed that *SlATG7* was highly expressed in stamens and carpels in tomatoes (Fig. 7a).

Ten downregulated transgenic plants were obtained to study the functions of the *SlATG7* gene in tomatoes. The RT-qPCR expression analysis showed the *SlATG7* decreased significantly in lines 4, 25, and 37, which were further analysed (Fig. S5, see online supplementary material). The homozygous transgenic lines (RNAi-SlATG7) showed a noticeable decrease in seed number compared with the WT plants (Fig. 7b).

Pollen viability of RNAi-SlATG7 plants was tested using I_2_-KI staining, and results revealed that downregulation of *SlATG7* decreased the pollen viability in tomato anthers (Fig. 7c). Then the pollen germination was investigated. As shown in Figure 7d, the germination rate of WT pollen was about 90%, while only 60%–70% in transgenic plants.

The SEM analysis showed that the RNAi-SlATG7 pollens were shriveled and irregular in shape compared with the WT pollens (Fig. 7e). Analysis of the semi-thin section indicated the RNAi-SlATG7 tapetum still had a complete cell morphology at the binuclear microspore stage (Fig. 7f). However, the WT tapetum disappeared utterly at the binuclear microspore stage (Fig. 7f). The combined results indicated that downregulation of *SlATG7* affected the tapetum degeneration in tomato anthers.

### Downregulation of SlATG7 inhibits autophagy in the tapetum

**Figure 8:**
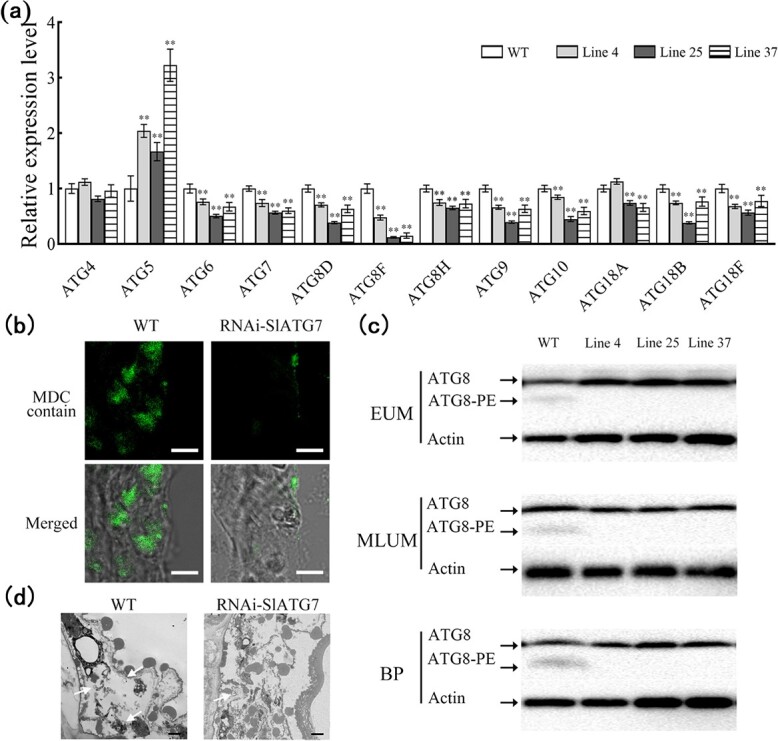
Analysis of autophagy in RNAi-SlATG7 and WT anther. **a** Expression levels of *ATGs* in RNAi-SlATG7 and WT anthers. The expression levels were determined by RT-qPCR. Data are means ± SD (six biological replicates). **b** Autophagosomes stained with MDC in the tapetum of RNAi-SlATG7 and WT plants. The tissue was induced with exogenous rapamycin. Scale bars = 5 μm. **c** ATG8 protein levels in RNAi-SlATG7 and WT anthers. ATG8-PE and ATG8 are the lipidated and nonlipidated ATG8 forms, respectively. Internal control is actin. EUM, MLUM, and BP represent the stages of early uninucleate microspore, middle, and later uninucleate microspore, and binucleate pollen. **d** TEM observation of autophagosomes in the tapetum of RNAi-SlATG7 and WT plants. The autophagosomes are indicated by white arrows. Scale bars = 1 μm. Asterisks represent significant differences (Student’s *t*-test, ^*^P < 0.05 and ^**^P < 0.01).

We further analysed the autophagy process in the anthers of RNAi-SlATG7 plants. The autophagy-related genes were analysed by RT-qPCR, and most of the autophagy-related genes were repressed in RNAi-SlATG7 lines compared with the WT plants (Fig. 8a). Then the MDC staining was used to detect autophagic activity in the RNAi-SlATG7 anthers. Low fluorescent signals were observed in the RNAi-SlATG7 tapetum, while strong fluorescent signals were observed in the WT plants (Fig. 8b).

To prove this result, WB was carried out to analyse the ATG8-PE formation in anthers. Accumulation of ATG8-PE was detected in WT anthers using the anti-ATG8a antibody, but the ATG8-PE band was hardly detected in RNAi-SlATG7 anthers (Fig. 8c). Then TEM was used to study the occurrence of autophagosomes in RNAi-SlATG7 anthers under the rapamycin treatment. The autophagosome vesicles of RNAi-SlATG7 anthers were hardly detected, while the autophagosome vesicles in the WT anthers were easily recognized (Fig. 8d). The data strongly supported that the downregulation of the *SlATG7* gene inhibited the autophagy in tomato anthers.

## Discussion

### Tapetum degradation affects pollen development in anther

The early or delayed tapetum degradation will cause abnormal growth of pollen [1, 15, 16]. Studies have found that several MYB transcription factors participate in pollen development by affecting the degradation process of tapetum [3, 6, 20]. Our study found that downregulation of the *SlMYB72* gene inhibited the degradation of the anther tapetum in tomatoes. The pollen of RNAi-SlMYB72 plants showed abnormal morphology and reduced viability (Fig. 2). Overexpression of the *SlMYB72* gene promoted the tapetum degradation and led to abnormal pollen development (Fig. 5). Downregulation of the *SlATG7* gene delayed the anther tapetum’s degradation, resulting in abnormal pollen development and reduced pollen viability (Fig. 3). The results prove that the *SlMYB72* and *SlATG7* genes can affect the tapetum degradation and pollen development, and the tapetum degradation plays important role in pollen development. The inhibition of the tapetum degradation will seriously interfere with the normal development of pollen.

### Autophagy is essential for pollen development in tomatoes

Studies have provided evidence that autophagy plays an important role in the degradation of anther tapetum and pollen development [32, 34–36]. This study showed that downregulation of the *SlMYB72* gene in tomatoes inhibited the expression of autophagy genes in tomato anthers. MDC staining and WB revealed that the autophagy in anthers of RNAi-SlMYB72 plants was significantly inhibited during the mononuclear microspore stage. The number of autophagosomes was decreased in the tapetum cells of the transgenic plants (Fig. 4). The results demonstrate the autophagy defect results in delayed tapetum degradation and abnormal pollen development in RNAi-SlMYB72 anthers, which are consistent with the results of Kurusu *et al.* [35]. In the OsATG7 mutant, the tapetum autophagy is inhibited, and the degradation of the tapetum layer is hindered, which eventually leads to pollen abortion.

We studied the function of SlATG7, which is homologous to OsATG7, in tomato pollen development. The MDC, WB, and anatomy results showed that downregulation of the *SlATG7* gene in tomatoes inhibited the autophagy occurrence and delayed tapetum degradation in anther. The RNAi-SlATG7 plants exhibited abnormal pollen morphology and reduced pollen viability (Fig. 7). The results indicate that the functions of the tomato *SlATG7* gene in pollen development are relatively consistent with that of rice *OsATG7* reported from Kurusu *et al.* [35]. The autophagy gene *ATG7* in rice and tomato affects anther tapetum degradation and pollen development under normal conditions. Arabidopsis ATG mediates pollen development at high temperatures but isn’t functional under normal conditions [36]. The Arabidopsis *ATG7* mutant undergoes normal embryogenesis, germination, and seedling development under normal conditions, demonstrating that the *ATG7* gene is not essential in the pollen development of Arabidopsis [38]. In tobacco, the downregulation of ATG7 affects the autophagy process in the anther but does not affect pollen development. These results indicate that the function of ATG7 in pollen development has functional differences among different species. The precise genomic editing of the *ATG* genes in different species will be carried out to identify the gene functions in tapetum degradation and pollen development in future research.

### Transcriptional regulation of *SlATG7* by SlMYB72

Under abiotic stress, the autophagy and *ATG* genes are transcriptionally regulated in many plants, such as tomato, pepper, rice, wheat, and Arabidopsis [39–47]. Some TFs have been reported to transcriptionally regulate autophagy and *ATG* genes. Heat shock factor 1A (HsfA1a) is involved in drought tolerance through regulating autophagy by directly transcriptional regulation of the *ATG10* and *ATG18f* genes in tomatoes [44]. WRKY DNA-binding protein 33 (WRKY33) functions as a possible autophagy modulator by regulating several *ATG* genes under heat stress in Arabidopsis [48]. TGA motif-binding protein 9 modulates the autophagy and *ATG* gene expression by direct binding to their promoters under the stress conditions of sucrose starvation and osmosis in Arabidopsis [49]. Until now, the transcriptional regulation of the *ATG* gene by TF is found only in abiotic stress conditions. In the present study, downregulation of *SlMYB72* decreased the expressions of *ATG* genes, while overexpression of *SlMYB72* increased the expression of some ATG genes (Figs 4 and 5). Downregulation of *SlMYB72* also inhibited the autophagy in tomato anther tapetum. The results of RT-qPCR showed that *SlMYB72* and *SlATG7* had similar expression patterns in anther developmental stage (Figs 3 and 6), indicating that SlMYB72 may directly regulate the expression of *SlATG7*. The EMSA and ChiP-qPCR confirmed that SlMYB72 could directly target the ACCAAC/ACCAAA motif of the *SlATG7* gene. Our research with the transient dual-luciferase assay found that SlMYB72 can transcriptionally activate the *SlATG7* expression (Fig. 6). Downregulation of *SlATG7* affected the tapetum degradation and pollen development in tomatoes. Our data indicate that SlMYB72 transcriptionally regulates the autophagy and *SlATG7* gene during pollen development in tomatoes. Our study provides a new cue that MYB TF can transcriptionally regulate autophagy and ATG genes during plant development. Further study directly targeting SlMYB72 to other ATG genes is necessary to understand the transcriptional regulation of autophagy in the plant.

In summary, both SlMYB72 and SlATG7 affect tapetum degradation and pollen development in tomatoes. SlMYB72 affects the autophagy process in the pollen tapetum of tomatoes. SlMYB72 directly targets the *SlATG7* gene and actives its expression. SlATG7 also affects the autophagy process, tapetum degradation, and pollen development in tomatoes. The present study demonstrates that SlMYB72 affects the tapetum degradation and pollen development via transcriptional activation of *SlATG7* and autophagy in anthers. The results explore the roles of autophagy in tapetum degradation and pollen development and provide valuable candidate genes for the production of male sterility.

## Materials and methods

### Plant growth conditions

Tomato cultivar Micro-Tom (*Solanum lycopersicum* cv Micro-Tom) was used in this study. Seeds of Micro-Tom tomato were grown on soil in a standard greenhouse at 25 ± 2°C, 16-h-light/8-h-dark cycle, 250 mol min^−2^ s^−1^ intense light, and 60% humidity.

### Pollen viability and germination assays

Pollen assay was carried out as described previously [50]. Pollen grains were collected from the anthers at full bloom flower and transferred to 2% 2,3,5-triphenyl-2 h-tetrazolium chloride (TTC) solution in the dark at 37°C for 15 min. Then the pollens were analysed with a light microscope. Inviable pollens were unstained, while viable pollens were stained in red. The pollen grains were stained with I_2_-KI solution for 5 min and examined under a light microscope. Viable pollen grains were stained in the dark. The staining experiments were repeated at least three times.

The germination experiment was performed according to the report from Gan *et al.* [51]. Mature pollens were placed on slides with a germination medium containing 0.015% w/v boric acid, 13% w/v sucrose, and 1% w/v phytagel in the dark at 25°C for 2 and 6 h.

### Fluorescence observation of pollen tube growth

Pistils (2 DAP) were fixed with 3:1 ethanol:glacial acetic acid overnight. The tissues were softened with 8 M sodium hydroxide for 2 d and stained for 3 h with 0.05% aniline blue. The tissues were mounted in a drop of 50% glycerin and observed with a Leica DMRXA epifluorescence microscope.

### Plasmid construction and plant transformation

For construction of the SlMYB72 overexpression vector, the ORF sequence of the *SlMYB72* gene was inserted into pLP100 containing the cauliflower mosaic virus (CaMV) 35S promoter, which has been described in Wu *et al.* [37]. To construct the RNAi-SlATG7 vector, a 300-bp conserved sequence of SlATG7 was cloned into pCAMIBA2301 containing the 35S promoter. The construction was transferred into *Agrobacterium tumefaciens* GV3101 and transgenic tomato plants were obtained using *A. tumefaciens*-mediated method as previously described [37]. Transgenic lines of T3 generations were screened by 1/2 Murashige & Skoog (MS) medium containing 80 mg·L^−1^ kanamycin. In this study, sequences of primers are shown in Supplemental Table S1 (see online supplementary material).

### GUS staining and RT-qPCR

For GUS staining, different tissues from *SlMYB72* promoter-*GUS* plant were placed in a GUS staining solution containing 10 mM EDTA and 0.1 M sodium phosphate buffer at 37°C for 24 h. Samples were fixed overnight in FAA solution, washed by ethanol, embedded in paraffin, and sectioned at 5 μm with a microtome. The sections were observed under a microscope.

The expression levels of *SlATG7* in different tissues were analysed with a public tomato database (http://tomexpress.toulouse.inra.fr/). For RT-qPCR analysis of *SlMYB72* and *SlATG7* expression, RNA was extracted from various tissues using RNeasy Plant Mini Kit (Qiagen, Chongqing, China). One μg of RNA was reverse transcribed using HiScriptII Q Select RT SuperMix (Vazyme Biotech). RT-qPCR was performed using the SYBR Premix ExTaq kit (TaKaRa). Gene expression levels were calculated from the ΔΔt values. Ubiquitin and actin genes were used as the internal standard to normalize the expression. The RT-qPCR was carried out with four biological replicates. Sequences of primers are shown in Table S1 (see online supplementary material).

### Electrophoretic mobility shift assays

A full-length coding sequence of *SlMYB7*2 was inserted into a pGEX-4 T-1 bacterial expression vector containing a glutathione S-transferase (GST) tag and the expression vector was transformed into *Escherichia coli* (*E. coli*) Rosetta (DE3) strain. The fusion protein was induced at 20°C by 0.5 mM isopropyl-β-D-thiogalactopyranoside (IPTG) in the dark and purified through a GST-tagged purification kit (Clontech). Probes containing the AC-rich motif sequence from the gene’s promoter were labeled using a lightshift chemiluminescent EMSA kit (Thermo Fisher Scientific). The unlabeled probe was used as a competitor and the AC-rich motif sequence was changed to AAAAAA sequence as the mutant probe in this experiment. The recombinant protein and probes (biotin-labeled, competitor, and mutated probes) were used to carry out the EMSA reaction according to the previously described [37]. The SlMYB72-GST bound probes were separated from the unbound probes by polyacrylamide gel electrophoresis.

### Dual-luciferase transient expression assay

*SlATG7* promoter was analysed using PlantCARE database (http://bioinformatics.psb.ugent.be/webtools/plantcare/html/). The dual-luciferase transient expression assay was carried out to analyse the interaction between SlMYB72 and *SlATG7* genes. The full-length coding sequence of *SlMYB72* was inserted into pGreenII 62-SK as an effector vector. Promoter sequence of *SlATG7* was inserted into pGreen 0800-LUC as a reporter vector. The reporter and effector plasmids were cotransfected into leaves of tobacco by *A. tumefaciens*-mediated transient transformation. LUC and REN activities were determined using a dual luciferase assay kit (Promega). The experiments were biologically repeated at least six times. Sequences of primers are shown in Table S1 (see online supplementary material).

### ChIP-qPCR assays

The ChIP-qPCR assay was performed according to the previous study [37]. Transgenic plants overexpressing 35S-SlMYB72-FLAG were used for the assay in this study. The immunoprecipitated DNA fragments were determined using RT-qPCR for examining the relative enrichment of the promoter fragment. The specific primers used are listed in Table S1 (see online supplementary material).

### Light microscopy and electron microscopy

For semi-thin sections, flowers at different development stages were fixed overnight in 4% glutaraldehyde, dehydrated in ethanol, and embedded in epoxy resin. The samples were sectioned at 0.5 μm, and analysed under light microscopy. For SEM analysis, pollens were isolated from flowers and observed under a Hitachi TM-1000. For TEM analysis, a FEI Tecnai T12 twin TEM was used to examine the anther tissues according to the method described by Yuan *et al.* [52].

### MDC staining

For MDC staining, tissues were induced with exogenous rapamycin, fixed in 4% paraformaldehyde, and washed in PBS buffer by three times. Tissues were stained with 0.2 mM monodansylcadaverine (MDC) for 30 min and sectioned with a freezing microtome. Sections were analysed under a Leica TCS SP2 laser confocal microscope (excitement at 405 nm and detection at 445 to 465 nm).

### Protein extraction and western blotting

The anther tissues of transgenic plants were ground in RIPA buffer and placed on ice for 35 min for protein extraction. The extracted proteins were added to the protein loading buffer, heated at 95°C, and separated on a 15% SDS-PAGE gel for WB. Agrisera rabbit anti-Atg8 antibody and Abcam anti-actin antibody were used in this assay.

### Accession numbers

Sequence numbers for *SlMYB72*, *ATG1, ATG2*, *ATG4*, *ATG5*, *ATG6*, *ATG7*, *ATG8D*, *ATG8F*, *ATG8H*, *ATG9*, *ATG10*, *ATG18A*, *ATG18B* and *ATG18F* are Solyc07g055000. Solyc09g011320, Solyc01g108160, Solyc01g006230, Solyc02g036380, Solyc05g050390, Solyc11g068930, Solyc10g006270, Solyc08g078820, Solyc01g068060, Solyc04g008630, Solyc09g047840, Solyc08g006010, Solyc07g006120, and Solyc12g005230.

## Acknowledgments

This work was supported by the National Natural Science Foundation of China (32172596), the Technology Innovation and Application Development Project in Chongqing (cstc2021jscx-cylhX0115), the Chongqing Talents Innovation Leading Talents Project (cstc2022ycjh-bgzxm0018), the Tianfu Scholar Program of Sichuan Province (Department of Human Resources and Social Security of Sichuan Province 2021-58), and the Fundamental Research Funds for the Central Universities (2021CDJZYJH-002), and The Graduate Research and Innovation Foundation of Chongqing, China (CYB22048). We thank Jie Ye (Huazhong Agricultural University), Jianye Chen (South China Agricultural University), and Jianfei Kuang (South China Agricultural University) for providing experimental instruction.

## Author contributions

W.D., M.W., H.L., and Z.L. designed the research. M.W., Q.Z., G.W., X.X., X.H., Z.G., and Y.C. carried out experiments and collected and analysed the data. W.D., M.W., H.L. wrote the manuscript.

## Data availability statement

All relevant data and figures in this study can be found within the article and its supporting materials.

## Conflict of interest

The authors declare that they have no conflicts of interest.

## Supplementary data

Supplementary data is available at *Horticulture Research* online.

## Supplementary Material

Web_Material_uhac286

## References

[1] Yan MY , Xie DL, Cao JJ et al. Brassinosteroid-mediated reactive oxygen species are essential for tapetum degradation and pollen fertility in tomato. *Plant J.* 2020;102:931–47. 31908046 10.1111/tpj.14672

[2] Jeong HJ , Kang JH, Zhao M et al. Tomato male sterile 1035 is essential for pollen development and meiosis in anthers. *J Exp Bot.* 2014;65:6693–709. 25262227 10.1093/jxb/eru389 PMC4246194

[3] Phan HA , Iacuone S, Li SF et al. The MYB80 transcription factor is required for pollen development and the regulation of tapetal programmed cell death in Arabidopsis thaliana. *Plant Cell.* 2011;23:2209–24. 21673079 10.1105/tpc.110.082651 PMC3160043

[4] Sun YJ , Hord CLH, Chen CB et al. Regulation of arabidopsis early anther development by putative cell-cell signaling molecules and transcriptional regulators. *J Integr Plant Biol.* 2007;49:60–8.

[5] Xing SP , Salinas M, Höhmann S et al. miR156-targeted and nontargeted SBP-box transcription factors act in concert to secure male fertility in Arabidopsis. *Plant Cell.* 2010;22:3935–50. 21177480 10.1105/tpc.110.079343 PMC3027167

[6] Zhang H , Liang W, Yang X et al. Carbon starved anther encodes a MYB domain protein that regulates sugar partitioning required for rice pollen development. *Plant Cell.* 2010;22:672–89. 20305120 10.1105/tpc.109.073668 PMC2861464

[7] Lemmon RJ , Brown RC. γ-Tubulin and microtubule organization during meiosis in the liverwort Ricciocarpus natans (Ricciaceae). *Am J Bot.* 2008;95:664–71. 21632391 10.3732/ajb.2007388

[8] Ye J , Xu M. Actin bundler PLIM2s are involved in the regulation of pollen development and tube growth in Arabidopsis. *Plant Physiol.* 2012;169:516–22. 10.1016/j.jplph.2011.11.015 22209219

[9] Cecchetti V , Altamura MM, Falasca G et al. Auxin regulates Arabidopsis anther dehiscence, pollen maturation, and filament elongation. *Plant Cell.* 2008;20:1760–74. 18628351 10.1105/tpc.107.057570 PMC2518247

[10] Cheng Y , Dai X, Zhao Y. Auxin biosynthesis by the YUCCA flavin monooxygenases controls the formation of floral organs and vascular tissues in Arabidopsis. *Genes Dev.* 2006;20:1790–9. 16818609 10.1101/gad.1415106 PMC1522075

[11] Chen LF , Yang D, Zhang Y et al. Evidence for a specific and critical role of mitogen-activated protein kinase 20 in uni-to-binucleate transition of microgametogenesis in tomato. *New Phytol.* 2018;219:176–94. 29668051 10.1111/nph.15150

[12] Hu L , Liang W, Yin C et al. Rice MADS3 regulates ROS homeostasis during late anther development. *Plant Cell.* 2011;23:515–33. 21297036 10.1105/tpc.110.074369 PMC3077785

[13] Yu SX , Feng QN, Xie HT et al. Reactive oxygen species mediate tapetal programmed cell death in tobacco and tomato. *BMC Plant Biol.* 2017;17:76. 28427341 10.1186/s12870-017-1025-3 PMC5399379

[14] Goldberg RB , Beals TP, Sanders PM. Anther development, basic principles and practical applications. *Plant Cell.* 1993;5:1217–29. 8281038 10.1105/tpc.5.10.1217 PMC160355

[15] Ferguson AC , Pearce S, Band LR et al. Biphasic regulation of the transcription factor ABORTED MICROSPORES (AMS) is essential for tapetum and pollen development in Arabidopsis. *New Phytol.* 2017;213:778–90. 27787905 10.1111/nph.14200 PMC5215365

[16] Wu HM , Cheun AY. Programmed cell death in plant reproduction. *Plant Mol Biol.* 2000;44:267–81. 11199388 10.1023/a:1026536324081

[17] Parish RW , Li SF. Death of a tapetum, a programme of developmental altruism. *Plant Sci.* 2010;178:73–89.

[18] Pérez-Martín F et al. Developmental role of the tomato mediator complex subunit MED18 in pollen ontogeny. *Plant J.* 2018;96:300–15. 30003619 10.1111/tpj.14031

[19] Zhang ZB , Zhu J, Gao JF et al. Transcription factor AtMYB103 is required for anther development by regulating tapetum development, callose dissolution and exine formation in Arabidopsis. *Plant J.* 2007;52:528–38. 17727613 10.1111/j.1365-313X.2007.03254.x

[20] Alonso-Peral MM , Li J, Li Y et al. The microRNA159-regulated GAMYB-like genes inhibit growth and promote programmed cell death in Arabidopsis. *Plant Physiol*. 2010;154:757–71. 20699403 10.1104/pp.110.160630 PMC2949021

[21] Aya K , Ueguchi-Tanaka M, Kondo M et al. Gibberellin modulates anther development in rice via the transcriptional regulation of GAMYB. *Plant Cell.* 2009;21:1453–72. 19454733 10.1105/tpc.108.062935 PMC2700530

[22] Millar AA , Gubler F. The Arabidopsis GAMYB-like genes, MYB33 and MYB65, are microRNA-regulated genes that redundantly facilitate anther development. *Plant Cell.* 2005;17:705–21. 15722475 10.1105/tpc.104.027920 PMC1069693

[23] Liu XY , Yang M, Liu X et al. A putative bHLH transcription factor is a candidate gene for male sterile 32, a locus affecting pollen and tapetum development in tomato. *Hortic Res.* 2019;6:88. 31666957 10.1038/s41438-019-0170-2 PMC6804878

[24] Signorelli S , Tarkowski ŁP, van den Ende W et al. Linking autophagy to abiotic and biotic stress responses. *Trends Plant Sci.* 2019;24:413–30. 30824355 10.1016/j.tplants.2019.02.001 PMC6475611

[25] Barros JAS , Cavalcanti JHF, Medeiros DB et al. Autophagy deficiency compromises alternative pathways of respiration following energy deprivation in arabidopsis thaliana. *Plant Physiol.* 2017;175:62–76. 28710132 10.1104/pp.16.01576 PMC5580740

[26] Chung T , Phillips AR, Vierstra DR. ATG8 lipidation and ATG8-mediated autophagy in Arabidopsis require ATG12 expressed from the differentially controlled ATG12A AND ATG12B. *Plant J.* 2010;62:483–93. 20136727 10.1111/j.1365-313X.2010.04166.x

[27] Phillips AR , Suttangkakul A, Vierstra RD. The ATG12-conjugating enzyme ATG10 is essential for autophagic vesicle formation in arabidopsis thaliana. *Genetic.* 2008;178:1339–53. 10.1534/genetics.107.086199 PMC2278079 18245858

[28] Chen QW , Soulay F, Saudemont B et al. Overexpression of ATG8 in arabidopsis stimulates autophagic activity and increases nitrogen remobilization efficiency and grain filling. *Plant Cell Physiol.* 2019;60:343–52. 30407574 10.1093/pcp/pcy214

[29] Minina EA , Moschou PN, Vetukuri RR et al. Transcriptional stimulation of rate-limiting components of the autophagic pathway improves plant fitness. *J Exp Bot.* 2018;69:1415–32. 29365132 10.1093/jxb/ery010 PMC6019011

[30] Masclaux-Daubresse C , Chen Q, Have M. Regulation of nutrient recycling via autophagy. *Curr Opin Plant Biol.* 2017;39:8–17. 28528166 10.1016/j.pbi.2017.05.001

[31] Su WL , Bao Y, Yu X et al. Autophagy and its regulators in response to stress in plants. *Int J Mol Sci.* 2020;21:8889. 33255241 10.3390/ijms21238889 PMC7727659

[32] Singh SP , Pandey T, Srivastava R et al. BECLIN1 from arabidopsis thaliana under the generic control of regulated expression systems, a strategy for developing male sterile plants. *Plant Biotechnol J.* 2010;8:1005–22. 21050365 10.1111/j.1467-7652.2010.00527.x

[33] Singh SP , Singh SP, Pandey T et al. A novel male sterility-fertility restoration system in plants for hybrid seed production. *Sci Rep.* 2015;5:11274. 26073981 10.1038/srep11274 PMC4466886

[34] Hanamata S , Kurusu T, Kuchitsu K. Roles of autophagy in male reproductive development in plants. *Front Plant Sci.* 2014;5:457. 25309556 10.3389/fpls.2014.00457 PMC4163999

[35] Kurusu T , Koyano T, Hanamata S et al. OsATG7 is required for autophagy-dependent lipid metabolism in rice postmeiotic anther development. *Autophagy.* 2014;10:878–88. 24674921 10.4161/auto.28279 PMC5119067

[36] Dundar G , Shao Z, Higashitani N et al. Autophagy mitigates high-temperature injury in pollen development of Arabidopsis thaliana. *Dev Biol.* 2019;456:190–200. 31473188 10.1016/j.ydbio.2019.08.018

[37] Wu MB , Xu X, Hu X et al. SlMYB72 regulates the metabolism of chlorophylls, carotenoids, and flavonoids in tomato fruit. *Plant Physiol.* 2020;183:854–68. 32414899 10.1104/pp.20.00156 PMC7333684

[38] Zhao P , Zhou XM, Zhao LL et al. Autophagy-mediated compartmental cytoplasmic deletion is essential for tobacco pollen germination and male fertility. *Autophagy.* 2020;16:2180–92. 31983274 10.1080/15548627.2020.1719722 PMC7751669

[39] Chen Y , Yang L, Feng C et al. Nano neodymium oxide induces massive vacuolization and autophagic cell death in non-small cell lung cancer NCI-H460 cells. *Biochem Biophys Res Commun.* 2005;337:52–60. 16185655 10.1016/j.bbrc.2005.09.018

[40] Liu HB , Wu F, Wu X et al. Differential effects of rapamycin on Bursaphelenchus xylophilus with different virulence and differential expression of autophagy genes under stresses in nematodes. *Acta Biochim Biophys Sin.* 2019;51:254–62. 30668628 10.1093/abbs/gmy172

[41] Luo M , Cheng K, Xu Y et al. Plant responses to abiotic stress regulated by histone deacetylases. *Front Plant Sci.* 2017;8:2147. 29326743 10.3389/fpls.2017.02147 PMC5737090

[42] Pei D , Zhang W, Sun H et al. Identification of autophagy-related genes ATG4 and ATG8 from wheat (Triticum aestivum L.) and profiling of their expression patterns responding to biotic and abiotic stresses. *Plant Cell Rep.* 2014;33:1697–710. 24996626 10.1007/s00299-014-1648-x

[43] Perez-Martin M , Perez-Perez ME, Lemaire SD et al. Oxidative stress contributes to autophagy induction in response to endoplasmic reticulum stress in Chlamydomonas reinhardtii. *Plant Physiol.* 2014;166:997–1008. 25143584 10.1104/pp.114.243659 PMC4213124

[44] Wang Y , Cai S, Yin L et al. Tomato HsfA1a plays a critical role in plant drought tolerance by activating ATG genes and inducing autophagy. *Autophagy.* 2015;11:2033–47. 26649940 10.1080/15548627.2015.1098798 PMC4824577

[45] Xia KF , Liu T, Ouyang J et al. Genome-wide identification, classification, and expression analysis of autophagy-associated gene homologues in rice (Oryza sativa L.). *DNA Res.* 2011;18:363–77. 21795261 10.1093/dnares/dsr024 PMC3190957

[46] Zhai YF , Guo M, Wang H et al. Autophagy, a conserved mechanism for protein degradation, responds to heat, and other abiotic stresses in Capsicum annuum L. *Front Plant Sci.* 2016;7:131. 26904087 10.3389/fpls.2016.00131 PMC4746239

[47] Zhou J , Zhang Y, Qi J et al. E3 ubiquitin ligase CHIP and NBR1-mediated selective autophagy protect additively against proteotoxicity in plant stress responses. *PLoS Genet.* 2014;10:e1004116. 24497840 10.1371/journal.pgen.1004116 PMC3907298

[48] Zhou J , Wang J, Zheng Z et al. Characterization of the promoter and extended C-terminal domain of Arabidopsis WRKY33 and functional analysis of tomato WRKY33 homologues in plant stress responses. *J Exp Bot.* 2015;66:4567–83. 25969555 10.1093/jxb/erv221 PMC4507763

[49] Wang P , Nolan TM, Yin Y et al. Identification of transcription factors that regulate ATG8 expression and autophagy in Arabidopsis. *Autophagy.* 2020;16:123–39. 30909785 10.1080/15548627.2019.1598753 PMC6984607

[50] Sulusoglu M , Cavusoglu A. *In vitro* pollen viability and pollen germination in cherry laurel (*Prunus laurocerasus* L.). *Sci World J.* 2014;2014:657123. 10.1155/2014/657123 PMC4227381 25405230

[51] Gan ZY , Feng Y, Wu T et al. Downregulation of the auxin transporter gene SlPIN8 results in pollen abortion in tomato. *Plant Mol Biol.* 2019;99:561–73. 30734902 10.1007/s11103-019-00836-8

[52] Yuan YJ , Xu X, Gong Z et al. Auxin response factor 6A regulates photosynthesis, sugar accumulation, and fruit development in tomato. *Hortic Res.* 2019;6:85. 31645946 10.1038/s41438-019-0167-x PMC6804849

