# Supplementary material for: SlMYB72 affects pollen development by regulating autophagy in tomato
Source: Hortic Res. 2022 Dec 29;10(3):uhac286. doi: 10.1093/hr/uhac286 (PMC10015339; doi:10.1093/hr/uhac286)
Supplement: Web_Material_uhac286 [file web_material_uhac286.docx]

**SlMYB72 affects pollen development by regulation of autophagy in tomato**

Mengbo Wu^1,2^, Guanle Wu^1,2^, Lu Zhang^3^, Xin Xu^1,2^, Xiaowei Hu^1,2^, Zehao Gong^1,2^, Zhengguo Li^1,2^, Wei Deng^1,2^*

^1^ Key Laboratory of Plant Hormones and Development Regulation of Chongqing, School of Life Sciences, Chongqing University, Chongqing 400044, China

^2^ Center of Plant Functional Genomics, Institute of Advanced Interdisciplinary Studies, Chongqing University, 401331 Chongqing, China

^3^ Department of Horticulture and Landscape Architecture, Oklahoma State University, Stillwater, OK 74078, USA

**Supplemental Figure S1.** The expression of *SlMYB72* in transgenic lines.

**Supplemental Figure S2.** Sequences and phylogenetic analysis of SlATG7 protein.

**Supplemental Figure S3.** The expression of *SlATG7* in the OE-SlMYB72 transgenic plant.

**Supplemental Figure S4.** Online analysis of the expression of *SlATG7* in tomato.

**Supplemental Figure S5.** The expression of *SlATG7* in transgenic lines.

**Supplemental Table S1.** List of oligonucleotide primers.

**Supplementary Figures**


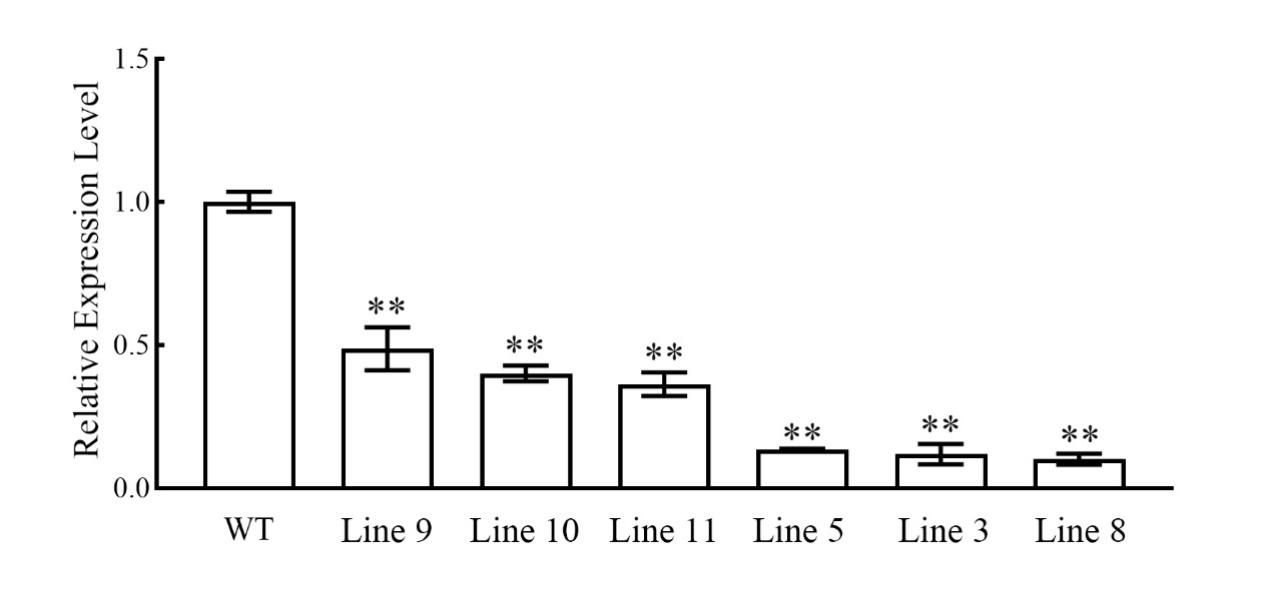


**Figure S1.** RT-qPCR analysis of the expression of *SlMYB72* in transgenic lines. The data represent means ± SD of four biological replicates. The relative expression levels of *SlMYB72* were compared between the transgenic and WT plants. Asterisks indicate significant differences between the transgenic and WT plants (*P<0.05 and **P<0.01), as determined by Student’s t test.


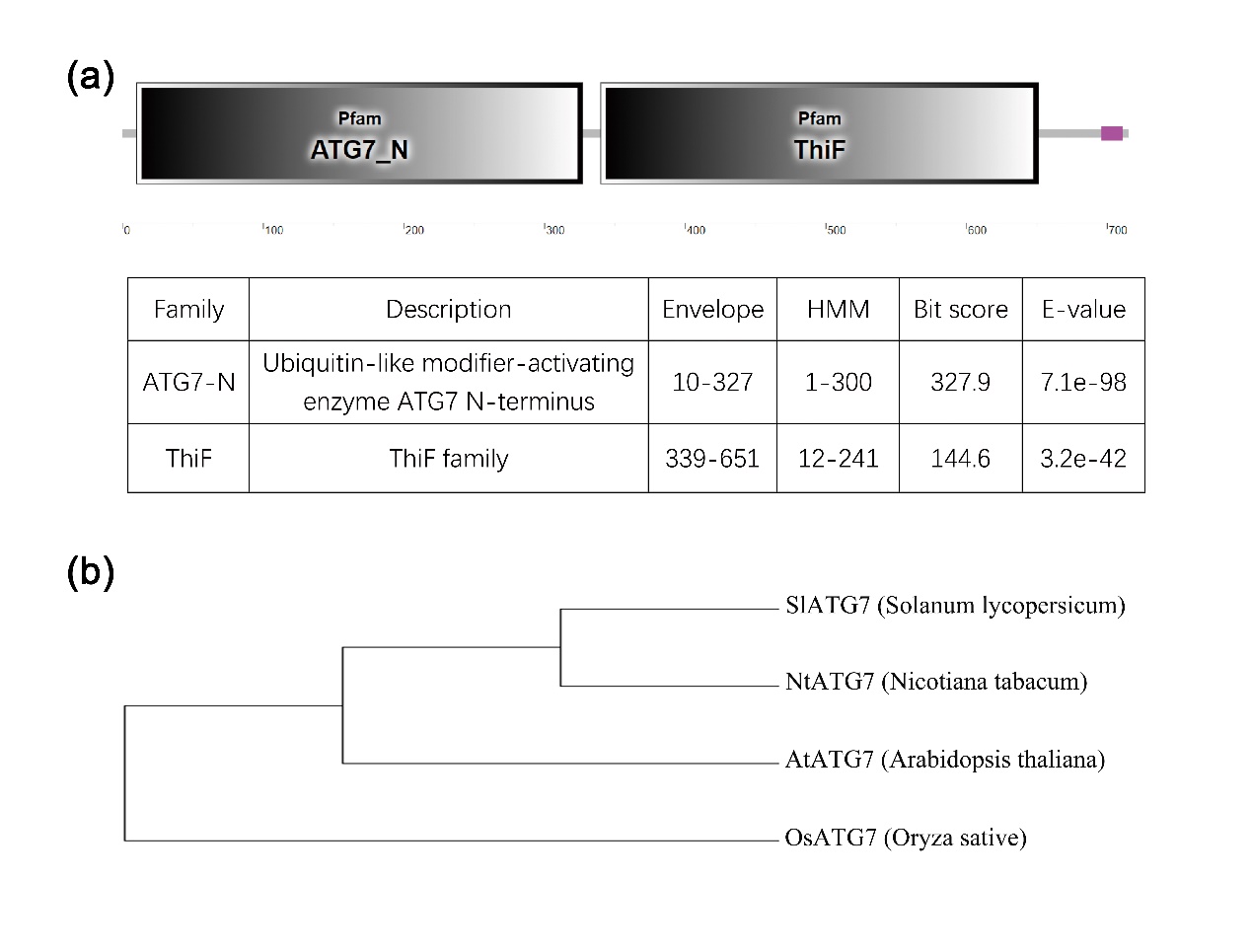


**Figure S2.** Sequences and phylogenetic analysis of SlMYB72. (**a**) Sequence analysis of SlATG7. The protein sequence of the SlATG7 was submitted in a public database (http://smart.embl-heidelberg.de/smart/show_motifs.pl) to analyze the conserved domain. ATG7-N is the N-terminal domain of ubiquitin-like modifier-activating enzyme ATG7. ThiF domain is a NAD/FAD-binding fold found in ubiquitin activating E1 family and members of the bacterial ThiF/MoeB/HesA family. (**b**) SlATG7 phylogenetic analysis. The phylogenetic tree was constructed using the Neighbor-Joining method.


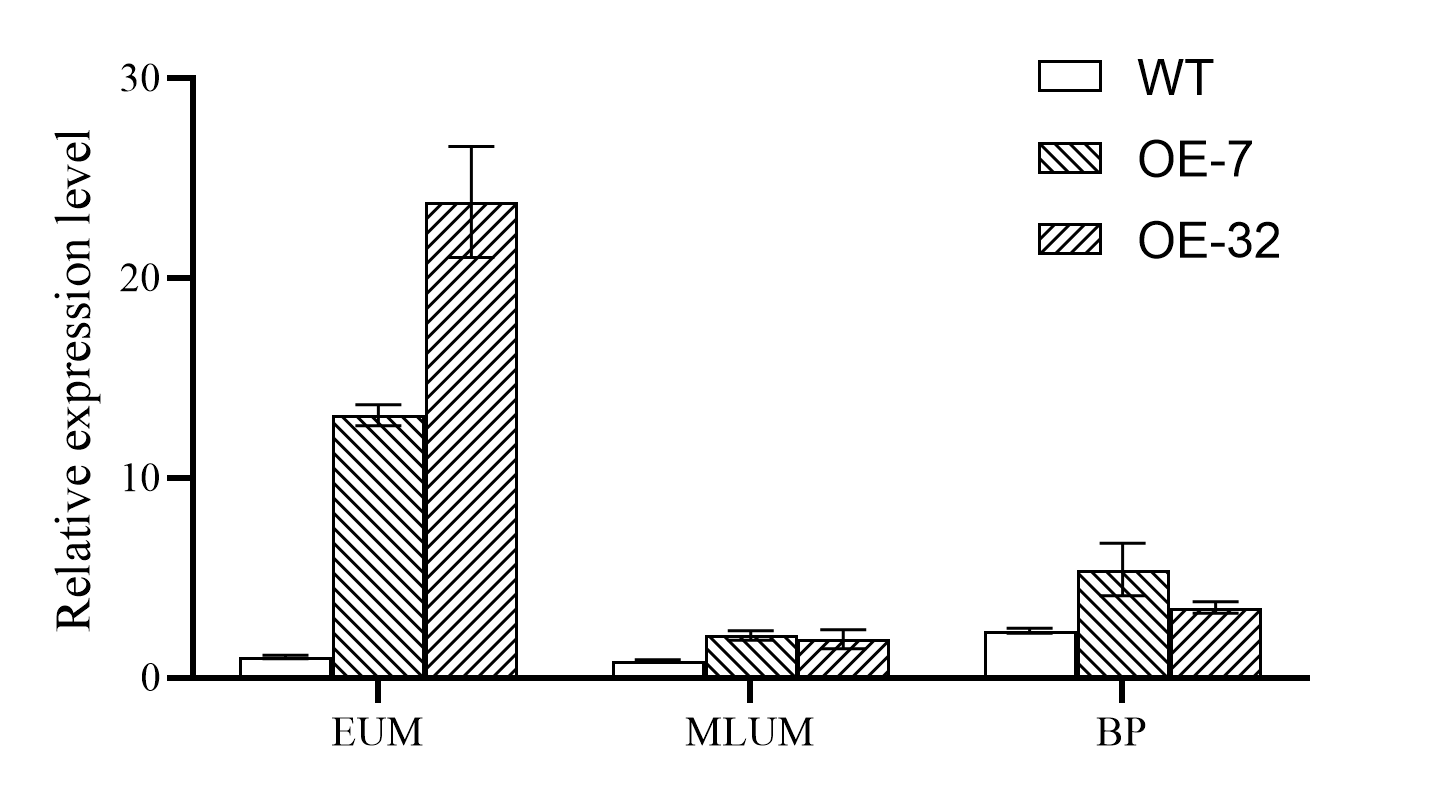


**Figure S3.** RT-qPCR analysis of the expression levels of of *SlATG7* in the OE-SlMYB72 transgenic plant at different development stages. EUM, MLUM, and BP represent the stages of early uninucleate microspore, middle and later uninucleate microspore, and binucleate pollen. The data are means ± SD (four biological replicates). Asterisks represent significant differences between WT and RNAi-SlMYB72 plants (Student’s t-test, *P<0.05 and **P<0.01). Scale bar = 50 μm.


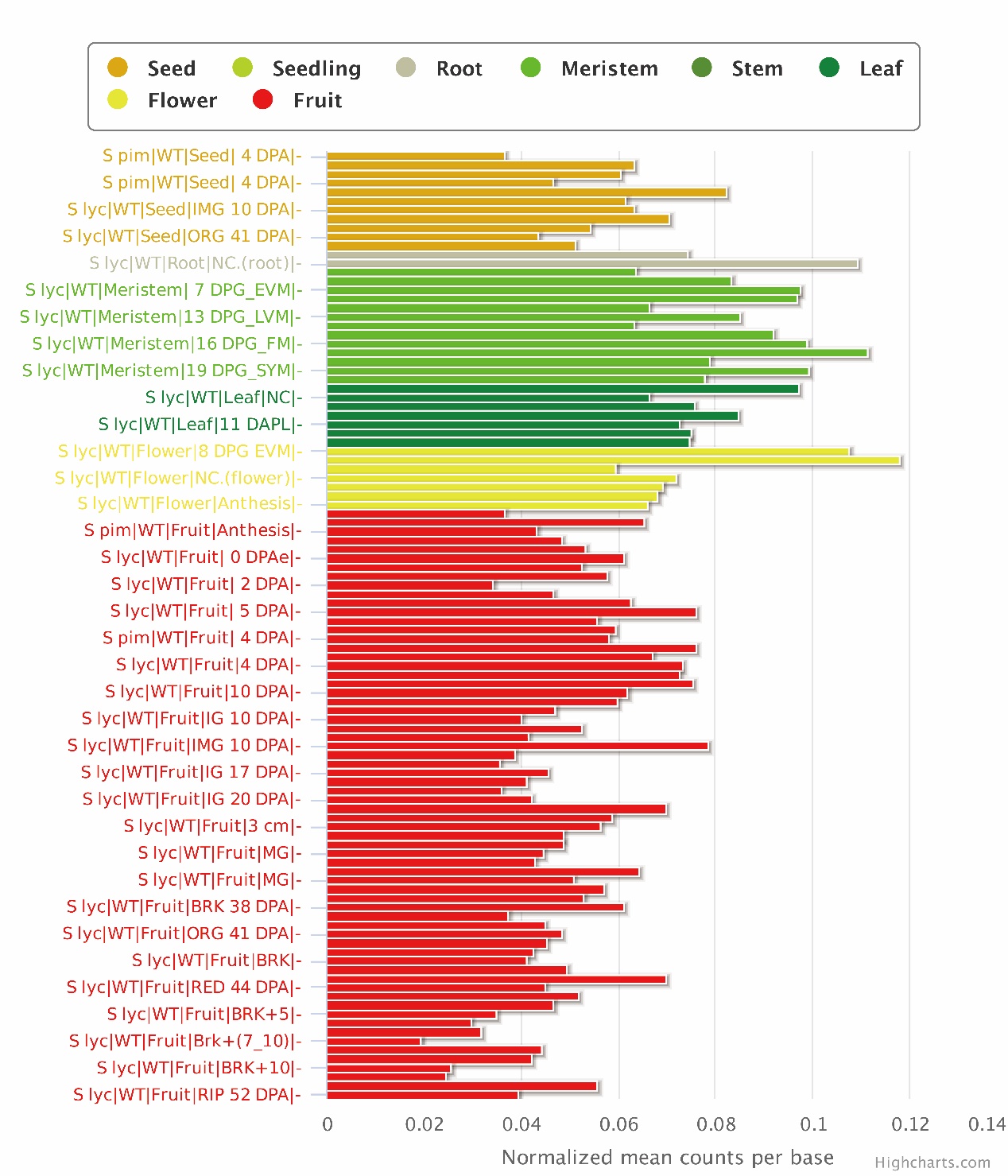


**Figure S4.** Analysis of SlATG7 expression in tomato plants using the online TomExpress platform.


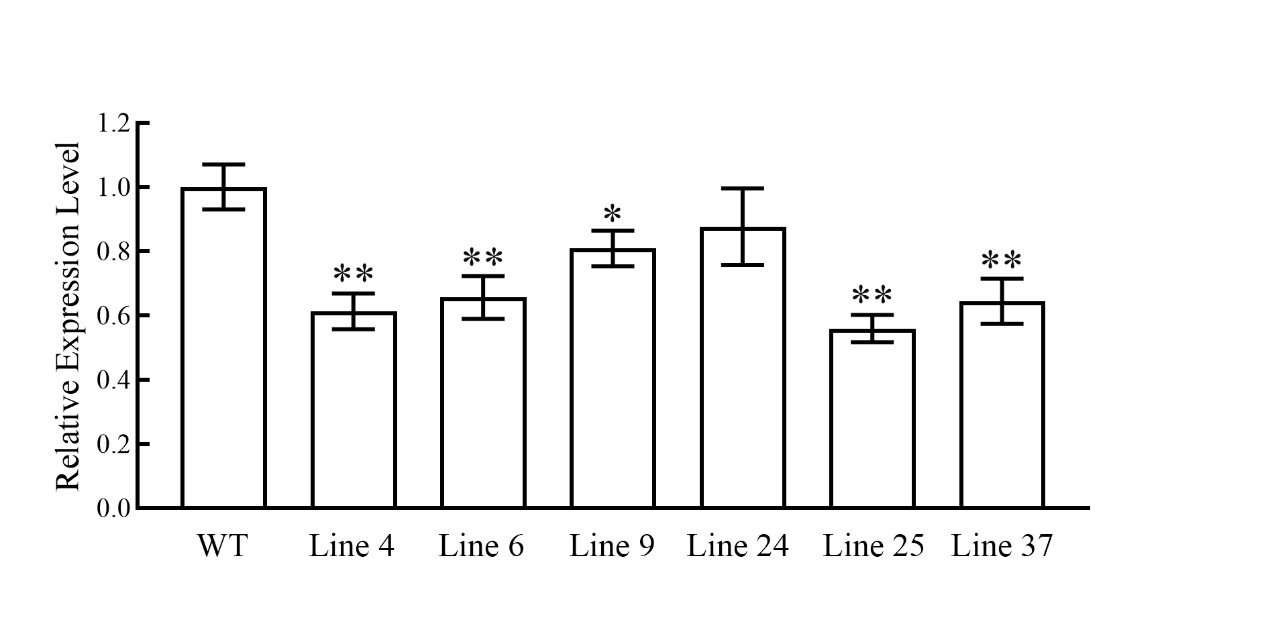


**Figure S5.** RT-qPCR analysis of the expression of *SlATG7* in the transgenic lines. The data represent means ± SD of four biological replicates. The relative expression levels of genes were compared between the RNAi-SlATG7 and WT plants. Asterisks indicate significant differences between the transgenic and WT plants (*P<0.05 and **P<0.01), as determined by Student’s t test.

**Supplementary Table.** List of oligonucleotide primers.

| Primers for vector construction | |  |
| --- | --- | --- |
| gene name | gene ID | primers sequence |
| RNAi-SlATG7 | Solyc11g068930 | F 5’-GGGGACAAGTTTGTACAAAAAAGCAG  GCTTAGAGTTTGCAAACTCTAATGATAACG-3’ |
|  |  | F 5’-GGGGACCACTTTGTACAAGAAAGCTGG  GTAGTTGTCATCATTTTCACTATCGTTA-3’ |
| pGreenII 62-SK-SlMYB72 | Solyc07g055000 | F 5’-TAGAACTAGTGGATCCATGGT  GAGAACAACTTGCTACG-3’ |
|  |  | F 5’-CGGTATCGATAAGCTTTTACAA  ATTA TAATGATCTA GATC-3’ |
| pGreenII 0800-SlATG7 | Solyc11g068930 | F 5’-TATAGGGCGAATTGGCTGATGG  AAGGGCAAAATTT-3’ |
|  |  | F 5’-TTGGCGTCTTCCATGGGGATAAA  ACTCGACCAACAA-3’ |
|  |  |  |
| Primers for EMSA | |  |
| gene name | gene ID | primers sequence |
| SlATG7-probe | Solyc11g068930 | F 5’-CATGGATAAAACTCGACCAACA  AAATCTTGAGCTTA-3’ |
|  |  | F 5’-TAAGCTCAAGATTTTGTTGGTC  GAGTTTTATCCATG-3’ |
| SlATG7-mutant probe | Solyc11g068930 | F 5’-CATGGATAAAACTCGAAAAAA  AAAATCTTGAGCTTA-3’ |
|  |  | F 5’-TAAGCTCAAGATTTTTTTTTTCG  AGTTTTATCCATG-3’ |
|  |  |  |
| Primers for CHIP | |  |
| gene name | gene ID | primers sequence |
| SlATG7-CHIP | Solyc07g055000 | F 5’-GCAAGATAGGTCAACGAACA-3’ |
|  |  | F 5’-GGTGCAAATTGAAGAATTGT-3’ |
|  |  |  |
| Primers for qRT-PCR | |  |
| gene name | gene ID | primers sequence |
| ATG1a | Solyc09g011320 | F 5’-AGTTCGGAAAGTC CCTCATC-3’ |
|  |  | F 5’-ATGATAGCAGAGGCA GAACG-3’ |
| ATG4 | Solyc01g006230 | F 5’-AATTGATCCCTCC TTGGCTA-3’ |
|  |  | F 5’-GATGTGGCAGAGCTA CGAGT-3’ |
| ATG5 | Solyc02g036380 | F 5’-TCAGATGGTGCTG AGATCAAG-3’ |
|  |  | F 5’-ATTGTTTACCACCCAT GCAA-3’ |
| ATG6 | Solyc05g050390 | F 5’-CCCATGCAGTCAA ACAATTC-3’ |
|  |  | F 5’-CCCTCATGCATTCAA GACAC-3’ |
| ATG7 | Solyc11g068930 | F 5’-ATTCAACGGCTAA CCGTACC-3’ |
|  |  | F 5’-CAAACTCAGCTTTGG CACAT-3’ |
| ATG8d | Solyc10g006270 | F 5’-AAGGCTGACAGAAGTGA-3’ |
|  |  | F 5’-ACTAACGAACTTGGGTGA-3’ |
| ATG8f | Solyc08g078820 | F 5’-GGCAATCATGTCTGCAATCT-3’ |
|  |  | F 5’-TCAAAGCTACAGTTCGCTCAG-3’ |
| ATG8h | Solyc01g068060 | F 5’-CGTGTTTGTGAAT AACACCTTG-3’ |
|  |  | F 5’-AGCACATGTAGAGGA ACCCA-3’ |
| ATG9 | Solyc04g008630 | F 5’-ATGTGCATCCTGA AATCGAA-3’ |
|  |  | F 5’-GCCTCTCGAAGAACA AGTCC-3’ |
| ATG10 | Solyc09g047840 | F 5’-GGAGAACCCTTGG CAATAGA-3’ |
|  |  | F 5’-TAGTCCCACATGGAT GCAAT-3’ |
| ATG18a | Solyc08g006010 | F 5’-CAGCGAGTTCACC ACTATCC-3’ |
|  |  | F 5’-TCCATCCAAGCCAAG AATTA-3’ |
| ATG18f | Solyc12g005230 | F 5’-TCCGAAGCAGAAC TCCAAAT-3’ |
|  |  | F 5’-AACCTCAGCCTCTCC ACGAC-3’ |
| ATG18b | Solyc07g006120 | F 5’-TTGAGGAGACAGCAACACCT-3’ |
|  |  | F 5’-TGTTCTGATGGTTGACGTTG-3’ |
